# IgSF11-mediated phosphorylation of pyruvate kinase M2 regulates osteoclast differentiation and prevents pathological bone loss

**DOI:** 10.1038/s41413-023-00251-2

**Published:** 2023-03-16

**Authors:** Hyunsoo Kim, Noriko Takegahara, Yongwon Choi

**Affiliations:** Department of Pathology and Laboratory Medicine, University of Pennsylvania Perelman School of Medicine, Philadelphia, PA 19104 USA

**Keywords:** Bone, Homeostasis

## Abstract

Osteoclasts are primary bone-resorbing cells, and receptor-activated NF-kB ligand (RANKL) stimulation is the key driver of osteoclast differentiation. During late-stage differentiation, osteoclasts become multinucleated and enlarged (so-called “maturation”), suggesting their need to adapt to changing metabolic demands and a substantial increase in size. Here, we demonstrate that immunoglobulin superfamily 11 (IgSF11), which is required for osteoclast differentiation through an association with the postsynaptic scaffolding protein PSD-95, regulates osteoclast differentiation by controlling the activity of pyruvate kinase M isoform 2 (PKM2). By using a system that directly induces the activation of IgSF11 in a controlled manner, we identified PKM2 as a major IgSF11-induced tyrosine-phosphorylated protein. IgSF11 activates multiple Src family tyrosine kinases (SFKs), including c-Src, Fyn, and HcK, which phosphorylate PKM2 and thereby inhibit PKM2 activity. Consistently, IgSF11-deficient cells show higher PKM2 activity and defective osteoclast differentiation. Furthermore, inhibiting PKM2 activities with the specific inhibitor Shikonin rescues the impaired osteoclast differentiation in IgSF11-deficient cells, and activating PKM2 with the specific activator TEPP46 suppresses osteoclast differentiation in wild-type cells. Moreover, PKM2 activation further suppresses osteoclastic bone loss without affecting bone formation in vivo. Taken together, these results show that IgSF11 controls osteoclast differentiation through PKM2 activity, which is a metabolic switch necessary for optimal osteoclast maturation.

## Introduction

Osteoclasts are multinucleated giant cells that are derived from hematopoietic progenitors of the monocyte/macrophage lineage.^1^ Osteoclast differentiation is driven and sustained primarily by RANKL,^2^ which is mainly produced by osteoblasts and osteocytes.^1,3,4^ During late-stage differentiation, osteoclasts become multinucleated and enlarged, suggesting a need to adapt to changing metabolic demands.^5,6^ However, the molecular mechanisms underlying the metabolic adaptation of osteoclasts are poorly understood.

Mammalian cells use the glycolytic pathway to oxidize glucose to meet the demands for energy production and anabolic processes, such as protein, lipid, and nucleic acid synthesis, and support proliferation and growth.^7–9^ Pyruvate kinases (PKs) catalyze the last step of glycolysis by transferring high-energy phosphate from phosphoenolpyruvate (PEP) to ADP to produce ATP and pyruvate.^10^ Pyruvate is subsequently reduced to lactate by lactate dehydrogenase (LDH) or is directed to the respiratory chain for high output of ATP. Furthermore, a reduction in PK enzymatic activity could result in the accumulation of glycolytic intermediates that are redirected toward biosynthesis. In mammals, four different PK isoforms exist. The *pkrl* gene encodes the PKL and PKR isoforms,^11^ and the *pkm* gene generates the M1 and M2 isoforms through alternative splicing.^12,13^ PKM1 is expressed in differentiated adult tissues. PKM2 is primarily expressed in proliferating cells, including fetal tissues and cancer cells. PKM1 has high constitutive enzymatic activity, whereas PKM2 is less active but can be allosterically activated by the upstream glycolytic metabolite fructose-1,6-bisphosphate (FBP).^14–16^ Importantly, inhibiting the enzymatic activity of PKM2 increases the fraction of glucose metabolites that are directed to anabolic pathways and then incorporated into macromolecule synthesis. This result is widely thought to provide a metabolic advantage to growing cells, including cancer cells.^9,17^

In this study, we demonstrate that immunoglobulin superfamily 11 (IgSF11) regulates osteoclast maturation by controlling the activity of PKM2. IgSF11 was originally reported to be a cell adhesion molecule (CAM),^18,19^ and it was subsequently revealed to be a component of the receptor- and signal transduction molecule-containing protein complex.^20^ IgSF11 is required for osteoclast differentiation and maturation through its association with the postsynaptic scaffolding protein PSD-95.^21^ Stimulation of IgSF11 induces the tyrosine phosphorylation of multiple intracellular proteins, and PKM2 was identified as a major IgSF11-induced tyrosine-phosphorylated protein. IgSF11 contributes to PKM2 tyrosine phosphorylation through multiple SFKs (c-Src, Fyn, and HcK), thereby inhibiting PKM2 activity. PKM2 inhibition ameliorates impaired osteoclast differentiation in IgSF11-deficient cells and further enhances osteoclast differentiation. In contrast, PKM2 activation suppresses osteoclast differentiation, protecting against pathological bone loss. These results show that IgSF11 controls osteoclast differentiation through PKM2 activity, which is a metabolic switch necessary for optimal osteoclast maturation.

## Results

### IgSF11 stimulation induces tyrosine phosphorylation of pyruvate kinase M2

**Fig. 1:**
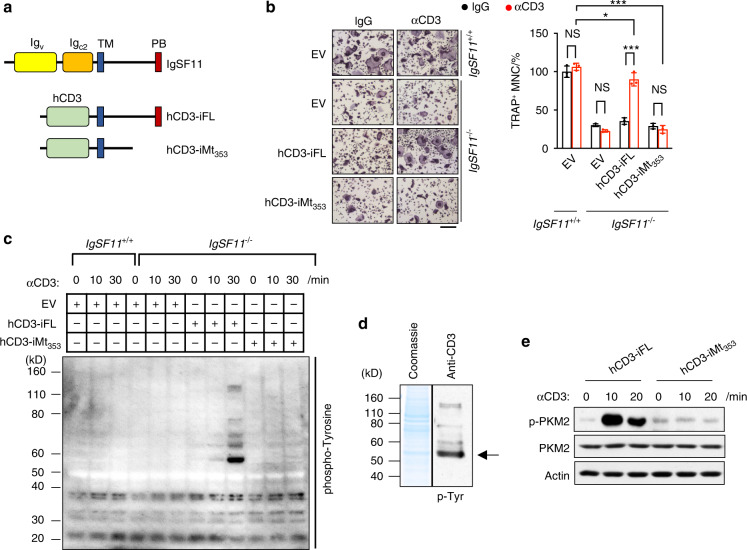
Stimulation of IgSF11 induces the phosphorylation of pyruvate kinase M2. **a** Schematics of IgSF11 (top), the human CD3 extracellular-transmembrane region fused to the full IgSF11 intracellular region (hCD3-iFL) (middle), and the IgSF11-Mt_353_ intracellular region (hCD3-iMt_353_) (bottom) chimeric proteins. The hCD3-iFL and hCD3-iMt_353_ mutants were used for retroviral transduction of IgSF11^−/−^ BMMs. All constructs were Flag-tagged at the C-terminus. TM; transmembrane domain, PB; PDZ binding domain. **b** IgSF11^−/−^ BMMs were retrovirally transduced with the indicated vectors, cultured with M-CSF + RANKL in the presence of anti-hCD3 antibody or control IgG and stained for TRAP. The frequency of TRAP^+^ multinucleated cells (3 nuclei or more per cell) is shown. Scale bars represent 100 μm. EV; empty vector. The data are presented as the means ± S.D. Each dot represents a technical replicate. **c** Tyrosine phosphorylation downstream of IgSF11. IgSF11^−/−^ BMMs were retrovirally transduced with the indicated vectors, cultured with M-CSF + RANKL for two days and then stimulated with anti-hCD3 antibodies for the indicated times. IgSF11^+/+^ BMMs that were transduced with an empty vector were used as a negative control. Western blotting was performed with the indicated antibodies. **d** IgSF11^−/−^ BMMs were retrovirally transduced with hCD3-iFL, cultured with M-CSF + RANKL for two days and then stimulated with anti-hCD3 antibodies for 30 min. Whole-cell lysates were passed through phosphoprotein affinity columns. The eluted fraction was separated by SDS–PAGE, visualized with Coomassie blue R-250 and/or immunoblotted with 4G10. The arrow indicates the protein band that was subjected to mass spectrometry. **e** IgSF11 induces the phosphorylation of PKM2. IgSF11^−/−^ BMMs were retrovirally transduced with the indicated vectors, cultured with M-CSF + RANKL for two days and then stimulated with anti-hCD3 antibodies for the indicated times. Western blotting was performed with the indicated antibodies. The results are representative of at least three independent experiments

We previously reported that IgSF11 regulates osteoclast differentiation and maturation through its interaction with PSD-95 via 75 C-terminal amino acids.^21^ To investigate the signaling mechanisms by which IgSF11 regulates osteoclast differentiation, we used an approach that specifically activated the cytoplasmic region of IgSF11 in a controlled manner via the generation of chimeric proteins that fuse the human CD3 (hCD3) extracellular-transmembrane region to the full-length IgSF11 intracellular region (hCD3-iFL) or the 75 C-terminal amino acid-deleted intracellular region (hCD3-iMt_353_) (Fig. 1a). We retrovirally transduced IgSF11-deficient (IgSF11^−/−^) bone marrow monocytes (BMMs) with hCD3-iFL or hCD3-iMt_353_. The cells were cultured with M-CSF + RANKL to generate osteoclasts in the presence of the control IgG or anti-hCD3 antibodies to cross-link surface hCD3 and stimulate the IgSF11 intracellular region. Consistent with a previous report, IgSF11^−/−^ BMMs that were transduced with the empty vector showed impaired osteoclast differentiation compared to IgSF11^+/+^ BMMs that were transduced with the empty vector (Fig. 1b). While the expression of hCD3-iFL in the presence of the control IgG showed no apparent effect compared to that of IgSF11^−/−^ BMMs that were transduced with the empty vector control, the addition of the anti-hCD3 antibody rescued the defective formation of TRAP^+^ multinucleated cells (Fig. 1b). However, stimulation with anti-hCD3 did not rescue IgSF11^−/−^ BMMs that were transduced with hCD3-iMt_353_. These results showed that the signaling induced by the chimeric hCD3-iFL receptor could rescue the defects in IgSF11^−/−^ BMMs to a level similar to that of the wild-type IgSF11 receptor, which also required the 75 C-terminal amino acids.

We then sought to identify the relevant downstream molecular events induced by the activation of the IgSF11 intracellular region. We prepared RANKL-treated IgSF11^−/−^ BMMs expressing hCD3-iFL (IgSF11^−/−^ hCD3-iFL) or hCD3-iMt_353_ (IgSF11^−/−^ hCD3-iMt_353_). These cells were stimulated with an anti-hCD3 antibody, and then western blotting was performed to examine whether serine/threonine- or tyrosine-mediated signaling pathways were activated. We found no apparent differences in overall serine/threonine phosphorylation between IgSF11^−/−^ hCD3-iFL and IgSF11^−/−^ hCD3-iMt_353_ cells (data not shown). In contrast, the anti-hCD3 antibody induced pronounced tyrosine phosphorylation of multiple proteins, particularly protein(s) approximately 55 kD in size and in IgSF11^−/−^ hCD3-iFL cells but not IgSF11^−/−^ hCD3-iMt_353_ cells (Fig. 1c).

To determine which protein(s) were tyrosine-phosphorylated by IgSF11 activation, we prepared total cell lysates from anti-hCD3 antibody-stimulated IgSF11^−/−^ hCD3-iFL cultures and passed them through phosphoprotein affinity columns. The purified proteins were separated by SDS–PAGE, and tyrosine phosphorylation was confirmed by an anti-panphosphotyrosine mAb (4G10) (Fig. 1d). The major band (M.W. ~55 kD) was then excised from a Coomassie blue-stained gel (Fig. 1d) and subjected to mass spectrometry. Three candidate proteins were identified: vimentin, tubulin beta 5, and PKM2 (Fig. S1). Interestingly, mass spectrometry analysis of IgSF11-activated proteins identified a phospho-peptide containing Y^105^ as one of the unique identifiers of PKM (Fig. S1). Therefore, to confirm that PKM2 (M.W. 57.8 kD) was a major tyrosine-phosphorylated protein in response to IgSF11 stimulation and that phosphorylation occurred at Y^105^, we used an anti-phospho-PKM2 (Y^105^)-specific mAb to examine lysates from IgSF11^−/−^ hCD3-iFL and IgSF11^−/−^ hCD3-iMt_353_ cultures. IgSF11 stimulation increased the tyrosine phosphorylation of PKM2 at Y^105^, which depended on the presence of the critical C-terminal region of IgSF11 (Fig. 1e). Because vimentin and tubulin beta 5 are both cytoskeletal proteins, they may have been identified due to their overabundance rather than because of their specific activation by IgSF11, and we could not detect tyrosine phosphorylation of vimentin in IgSF11^−/−^ hCD3-iFL cells after anti-hCD3 antibody stimulation (Fig. S1).

### IgSF11 signaling complexes induce PKM2 phosphorylation

**Fig. 2:**
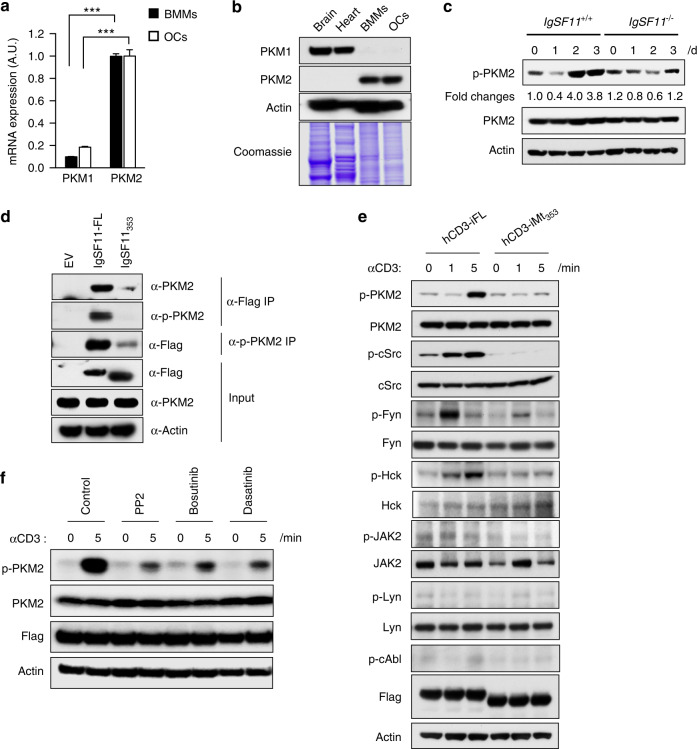
IgSF11 signaling complexes induce PKM2 phosphorylation. **a** Comparative expression of PKM isoform 1 and isoform 2 in wild-type cells. Total RNA was isolated from BMMs and osteoclasts and analyzed by Q-PCR. The data are presented as the means ± S.D. **b** Expression of PKM isoform 1 and isoform 2 in BMMs and osteoclasts. Adult mouse brains and hearts were used as controls. Whole cell/tissue lysates were used for western blotting with the indicated antibodies. Coomassie blue staining is shown as the protein loading control. **c** Phosphorylation of PKM2 during osteoclast differentiation. BMMs were cultured with M-CSF + RANKL for the indicated days, and whole cell lysates were used for western blotting with the indicated antibodies. **d** Coimmunoprecipitation of IgSF11 with PKM2. IgSF11^−/−^ BMMs were retrovirally transduced with the indicated vectors, lysed and immunoprecipitated with anti- Flag antibodies, and western blotting was performed with the indicated antibodies. **e** IgSF11 induces the phosphorylation of c-Src, Fyn and HcK. IgSF11^−/−^ BMMs were retrovirally transduced with the indicated vectors, cultured with M-CSF + RANKL for two days and then stimulated with anti-hCD3 antibodies for the indicated times. Western blotting was performed with the indicated antibodies. **f** Src family kinase inhibitors attenuated PKM2 phosphorylation. IgSF11^−/−^ BMMs were retrovirally transduced with hCD3-iFL, cultured with M-CSF + RANKL for two days and then stimulated with anti-hCD3 antibodies for the indicated times in the presence or absence of inhibitors (PP2; 4 μmol·L^–1^, bosutinib; 1 μmol·L^–1^, dasatinib; 1 μmol·L^–1^). Western blotting was performed with the indicated antibodies. The results are representative of at least three independent experiments

Since function of PKM2 in osteoclasts had not been previously addressed, we examined how this factor is regulated during osteoclast differentiation. Because the *Pkm* gene is alternatively spliced to generate transcripts encoding PKM1 or PKM2, we used specific primers for PKM1 and PKM2^22^ and performed qPCR analysis to identify the dominant isoform in osteoclasts. Individual cells generally express only one isoform at appreciable levels, and we found predominant expression of PKM2 in both BMMs and osteoclasts (Fig. 2a). Western blot analysis confirmed that the PKM2 protein was the predominantly expressed isoform in BMMs and osteoclasts, and its expression was similar in both cell types (Fig. 2b). The protein expression levels of PKM2 in IgSF11^−/−^ cells were comparable to those in IgSF11^+/+^ cells (Fig. 2c). These results suggest that PKM2 is the predominant isoform expressed in BMMs and osteoclasts. These findings also indicate that PKM2 expression is constant during osteoclast differentiation and is not affected by the absence of IgSF11.

The phosphorylation of Y^105^ negatively regulates PKM2 activity.^23^ We examined the phosphorylation of PKM2 at Y^105^ in BMMs and found that this signal continued to increase during RANKL-induced osteoclast differentiation in wild-type IgSF11^+/+^ cultures (Fig. 2c). In contrast, tyrosine phosphorylation of PKM2 did not increase in the absence of IgSF11 (Fig. 2c). These results suggest that PKM2 activity is gradually inhibited during osteoclast differentiation, especially in the late phase (Day 2 and Day 3), by the phosphorylation of Y^105^ and that this negative regulation is impaired in the absence of IgSF11.

We sought to identify the molecular mechanism by which IgSF11 phosphorylates PKM2. We previously showed that IgSF11 forms a signaling complex with PSD-95,^21^ and this signaling complex was required to induce PKM2 phosphorylation (Fig. 1e). We examined whether PKM2 associated with the signaling complex. IgSF11^−/−^ cells were retrovirally transduced with full-length IgSF11 (IgSF11-FL) or the C-terminal deletion mutant form of IgSF11 (IgSF11-Mt_353_), and then coimmunoprecipitation was performed. The association of PKM2 with IgSF11-FL was observed, regardless of its phosphorylation status (Fig. 2d). In contrast, an association between PKM2 and IgSF11-Mt_353_ was hardly observed (Fig. 2d). These results suggest that PKM2 is a component of the IgSF11 signaling complex. Given the absence of a kinase domain in IgSF11, we hypothesized that IgSF11 recruits tyrosine kinases to phosphorylate PKM2. IgSF11 interacts with PSD-95,^20,21^ which is a scaffold protein with multiple protein interaction domains. We looked for candidate kinases that could associate with PSD-95 or phosphorylate PKM2, such as c-Abl, JAK, c-Src, Fyn, and Lyn.^24–29^ We also examined whether IgSF11 activated these kinases in a stimulation-dependent manner by using IgSF11^−/−^ hCD3-iFL and IgSF11^−/−^ hCD3-iMt_353_ osteoclast differentiation cultures. We found that c-Src and Fyn were activated by anti-hCD3 antibody stimulation prior to the phosphorylation of PKM2, which was not observed when the IgSF11 intracellular region lacked the 75 critical C-terminal amino acids (Fig. 2e). Additionally, we found that Hck, a Src family kinase involved in osteoclast differentiation,^30^ was activated by anti-hCD3 antibody stimulation in a manner that was dependent on the 75 critical C-terminal amino acids of IgSF11 (Fig. 2e). To further examine the involvement of c-Src, Fyn, and HcK in IgSF11-mediated PKM2 phosphorylation, we used the inhibitors PP2 (targeting Src family kinases, including c-Src and Fyn),^31,32^ bosutinib (targeting Abl and Src family kinases, including c-Src),^33^ and dasatinib (targeting Abl and Src family kinases, including c-Src, Fyn, and HcK)^33,34^ in anti-hCD3 antibody-stimulated IgSF11^−/−^ hCD3-iFL cultures. We found a significant reduction in anti-hCD3 antibody-induced PKM2 phosphorylation in the presence of these inhibitors (Fig. 2f). These results suggest that multiple kinases, including c-Src, Fyn, and HcK, are activated in response to IgSF11 stimulation through the 75 C-terminal amino acids, leading to the phosphorylation of PKM2.

### The inhibition of PKM2 activity by IgSF11 is required for osteoclast differentiation

**Fig. 3:**
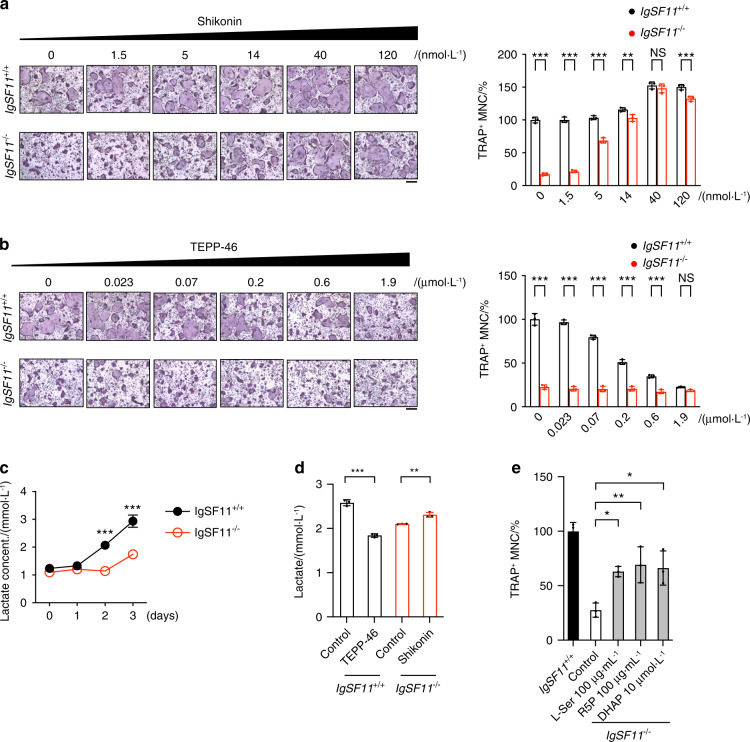
The inhibition of PKM2 activity by IgSF11 is required for osteoclast differentiation. **a** Effect of shikonin on osteoclast differentiation. IgSF11^+/+^ and IgSF11^−/−^ BMMs were cultured with M-CSF + RANKL for three days. The indicated dose of shikonin was added and incubated for three days. **b** Effect of TEPP46 on osteoclast differentiation. IgSF11^+/+^ and IgSF11^−/−^ BMMs were cultured with M-CSF + RANKL for three days. The indicated dose of TEPP46 was added and incubated for three days. TRAP staining and the frequency of TRAP^+^ multinucleated cells (3 nuclei or more per cell) are shown. Scale bars represent 100 μm. **c** Lactate production during osteoclast differentiation. IgSF11^+/+^ and IgSF11^−/−^ BMMs were cultured with M-CSF + RANKL for three days. The lactate concentration in the culture supernatants was measured daily. **d** Lactate production in response to the modulation of PKM2 activity. IgSF11^+/+^ and IgSF11^−/−^ BMMs were cultured with M-CSF + RANKL for three days. Control DMSO or TEPP46 was added to IgSF11^+/+^ cultures and incubated for three days. Control DMSO or shikonin was added to IgSF11^−/−^ cultures and incubated for three days. The lactate concentrations in the culture supernatants were measured. **e** Effect of glycolysis intermediates on osteoclast differentiation. IgSF11^+/+^ and IgSF11^−/−^ BMMs were cultured with M-CSF + RANKL for three days. The indicated dose of glycolysis intermediates was added to IgSF11^−/−^ cultures and incubated for three days. IgSF11^+/+^ cultures were used as a control. The frequency of TRAP^+^ multinucleated cells (3 nuclei or more per cell) is shown. The data are presented as the means ± S.D. Each dot represents a technical replicate. The results are representative of at least three independent experiments

We have shown that IgSF11 phosphorylates PKM2 at Y^105^ and that this modification decreases PKM2 activity. Thus, we hypothesized that the negative regulation of PKM2 activity by IgSF11 is required for optimal osteoclast differentiation. To address this point, IgSF11^+/+^ and IgSF11^−/−^ BMMs were cultured with M-CSF + RANKL in the presence of Shikonin, a specific PKM2 inhibitor,^35^ or TEPP46, a specific PKM2 activator.^36^ Shikonin restored osteoclast differentiation in IgSF11^−/−^ cultures in a dose-dependent manner and further enhanced osteoclast differentiation in IgSF11^+/+^ and IgSF11^−/−^ cultures (Fig. 3a). Conversely, when TEPP46 was added, there was a dose-dependent decrease in osteoclast differentiation (Fig. 3b). These results suggest that the lack of a decrease in PKM2 activity in IgSF11^−/−^ cells is the molecular defect that is responsible for decreased osteoclast formation in the absence of IgSF11.

We then sought to identify the role of IgSF11-mediated negative regulation of PKM2 during osteoclast differentiation. Given that the decrease in PKM2 activity regulates the rate-limiting step of glycolysis, which shifts glucose metabolism toward lactate production and facilitates the production of glycolytic intermediates that are needed for macromolecule biosynthesis, we hypothesized that the IgSF11-PKM2 pathway was required for biosynthetic and anabolic pathways. To address this point, IgSF11^+/+^ and IgSF11^−/−^ cells were cultured with M-CSF + RANKL to induce osteoclasts, and lactate production in the culture supernatants was measured. The accumulation of lactate during osteoclast differentiation was observed in IgSF11^+/+^ cultures, which was consistent with a previous report^37^ (Fig. 3c). In contrast, this accumulation was significantly attenuated in IgSF11^−/−^ cultures (Fig. 3c). In addition, we found that TEPP46 treatment inhibited lactate production in IgSF11^+/+^ cells, while Shikonin treatment enhanced the lactate production in IgSF11^−/−^ cells (Fig. 3d). These results suggested insufficient glycolysis in the absence of IgSF11, which could be attributed to impaired negative regulation of PKM2 activity.

TEPP46 treatment has been reported to downregulate glycolytic intermediates, including dihydroxyacetone phosphate (DHAP), which is a metabolite required for the biosynthesis of phospholipids and triacylglycerols.^38^ Additionally, TEPP46 treatment downregulates ribose 5-phosphate (R5P) and serine (Ser), which are involved in the biosynthesis of nucleotides and amino acids/phospholipids, respectively.^36,38^ When these metabolites were added to IgSF11^−/−^ cultures during osteoclast differentiation, osteoclast differentiation was partially but significantly restored (Fig. 3e). These results suggest that IgSF11-mediated negative regulation of PKM2 contributes to osteoclast differentiation by shunting glucose metabolites toward anabolic pathways.

### Proper PKM2 activity is required for bone homeostasis

**Fig. 4:**
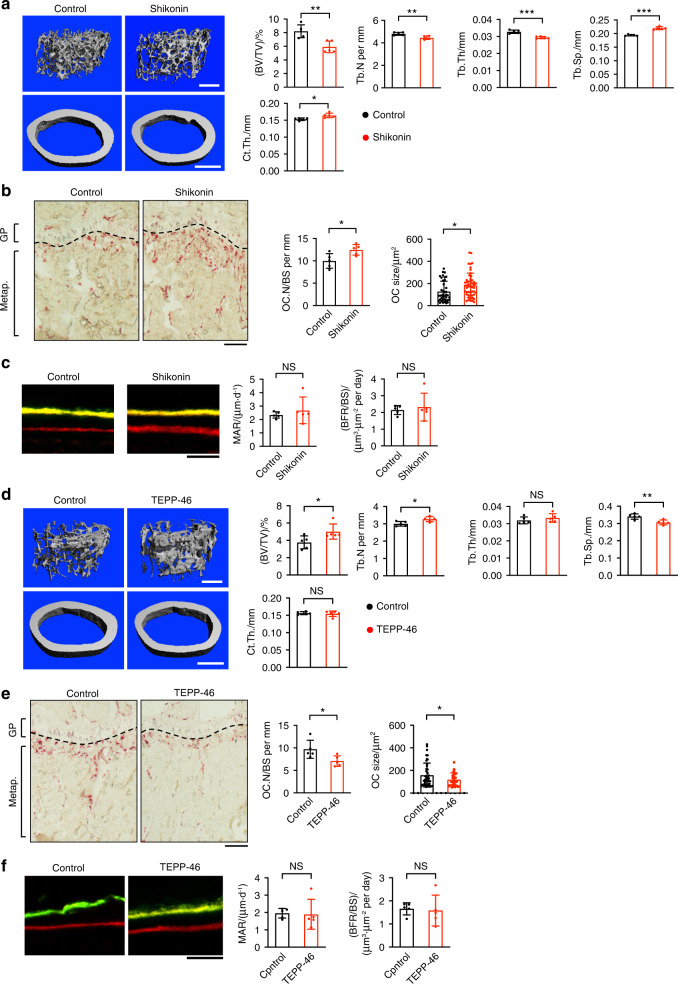
Proper PKM2 activity is required for bone homeostasis. **a**, **d** Representative microcomputed tomography (μCT) images of femurs from mice treated with shikonin (*n* = 5 mice per group) for 4 weeks (**a**) or TEPP46 (*n* = 5 mice per group) for 6 weeks (**d**). Bone volume per tissue volume (BV/TV), trabecular thickness (Tb.Th), trabecular number (Tb.N), trabecular spacing (Tb.Sp), bone mineral density (BMD), and cortical thickness (Ct.Th) are shown. Scale bars represent 0.5 mm. **b**, **e** Representative TRAP staining images of the tibias of mice treated with shikonin for 4 weeks (**b**) or TEPP46 for 6 weeks (**e**). The scale bar represents 200 μm. GP Growth plate, Metap. Metaphysis. **c**, **f** Representative dynamic histomorphometry of the tibias of mice treated with shikonin for 4 weeks (**c**) or TEPP46 for 6 weeks (**f**). The mineral apposition rate (MAR) and bone formation rate (BFR) are shown. The scale bar represents 50 μm. Each dot represents the result of a single mouse (*n* = 5 mice per group). Each dot in OC size represents the result of a single osteoclast. The data are presented as the means ± S.D

We then sought to identify the role of PKM2 activity in normal bone homeostasis in vivo. Wild-type mice were injected with Shikonin twice per week for four weeks, and representative 3D reconstructions of bone tissues were performed. Bone microstructure imaging by high-resolution microcomputed tomography (μCT) revealed a significant reduction in bone mass. This reduction was characterized by decreased bone indices, including trabecular bone volume per tissue volume (BV/TV), trabecular number (Tb.N), and trabecular thickness (Tb.th), and there was a concomitant increase in trabecular spacing (Tb.Sp.) in trabecular bone (Fig. 4a). Furthermore, TRAP-stained bone sections revealed significant increases in the number and size of osteoclasts on the bone surface compared to those in the control sections (Fig. 4b). Dynamic histomorphometry revealed a normal bone formation rate in mice that were treated with Shikonin (Fig. 4c). In contrast, mice injected with TEPP46 every two days for six weeks showed the opposite results. There was a significant increase in their bone mass, including increases in BV/TV and Tb.N and a decrease in Tb.Sp. in trabecular bone (Fig. 4d). Additionally, there was a significant decrease in the number (OC.N/BS) and size of osteoclasts on the bone surface (Fig. 4e). The bone formation rates in TEPP46-treated mice were comparable to those in untreated mice. (Fig. 4f). These results suggest that proper PKM2 activity is required to maintain bone homeostasis by regulating osteoclast differentiation without affecting coupled bone formation. Neither shikonin nor TEPP46 treatment changed body weight (Fig. S2). Additionally, no apparent abnormalities were observed in soft tissues (Fig. S2). These results support previous reports and indicate that mouse growth is largely unaffected by the modulation of PKM2 activity.^36,39^

### PKM2 activation attenuates osteoclastic bone loss

**Fig. 5:**
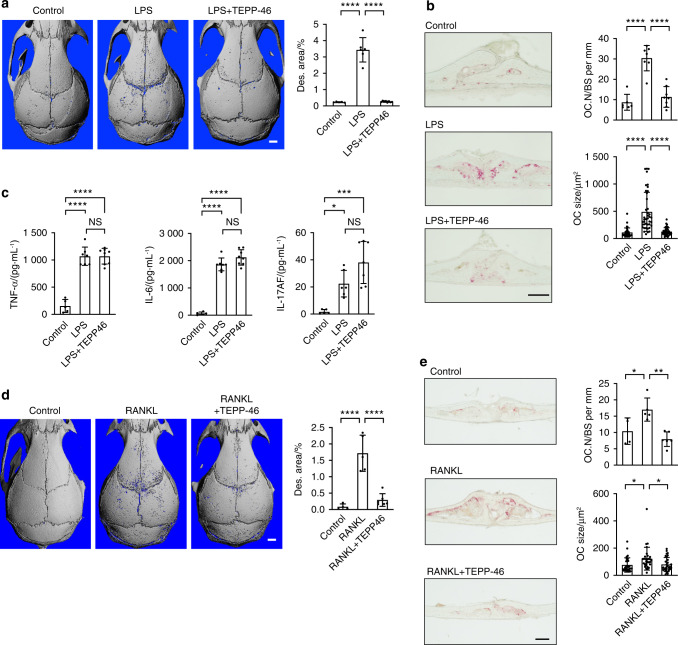
The activation of PKM2 attenuates LPS-induced osteoclastic bone loss. **a** Representative μCT images of mouse calvaria 5 days after LPS injection with or without TEPP46 (*n* = 5–6 mice per group). The scale bar represents 1 mm. Percentages of the bone destruction areas (Des. area) in calvaria are shown. **b** Representative TRAP staining images of calvarial coronal sections in (**a**). TRAP^+^ osteoclast size (OC size) and number (OC.N/BS) are shown. Scale bar represents 200 μm. **c** Serum levels of TNF-α, IL-6, and IL-17AF in the mice in (**a**) are shown. **d** Representative μCT images of mouse calvaria at 4 days after RANKL injection with or without TEPP46 are shown (*n* = 4–6 mice per group). The scale bar represents 1 mm. **e** Representative TRAP staining images of calvarial coronal sections in (**d**). The scale bar represents 200 μm. Each dot represents the result of a single mouse. Each dot in OC size represents the result of a single osteoclast. The data are presented as the means ± S.D

Having shown the importance of PKM2 activity in normal bone homeostasis, we next sought to investigate the effect of PKM2 activation on pathological bone destruction. We used a model of inflammatory bone loss in which LPS, which is a vital component of the outer membrane of gram-negative bacteria, is injected into the calvaria periosteum. This resulted in increased osteoclast numbers and localized bone resorption.^40^ Wild-type mice were injected with LPS with or without TEPP46. Five days later, the calvaria were subjected to high-resolution μCT imaging, followed by TRAP staining of histological sections of the sagittal suture. We found that LPS injection resulted in drastic bone destruction in the calvaria, and concurrent injection of TEPP46 completely inhibited LPS-induced bone destruction (Fig. 5a). TRAP staining revealed that LPS increased cell size and the number of TRAP^+^ osteoclasts compared to those in the control; however, these increases were completely abolished by concurrent injection of TEPP46 (Fig. 5b). Moreover, LPS injection significantly increased serum levels of inflammatory cytokines regardless of the presence of TEPP46 (Fig. 5c), suggesting that TEPP46 treatment did not affect inflammation. Additionally, we found that RANKL-induced inflammation-independent bone loss was significantly inhibited by concurrent injection of TEPP46 (Fig. 5d, e), which was consistent with the observation that TEPP46 targeted osteoclast differentiation without affecting inflammation. These results suggest that TEPP46-mediated PKM2 activation attenuated osteoclast differentiation and maturation without affecting inflammation during LPS-induced inflammatory bone loss.

**Fig. 6:**
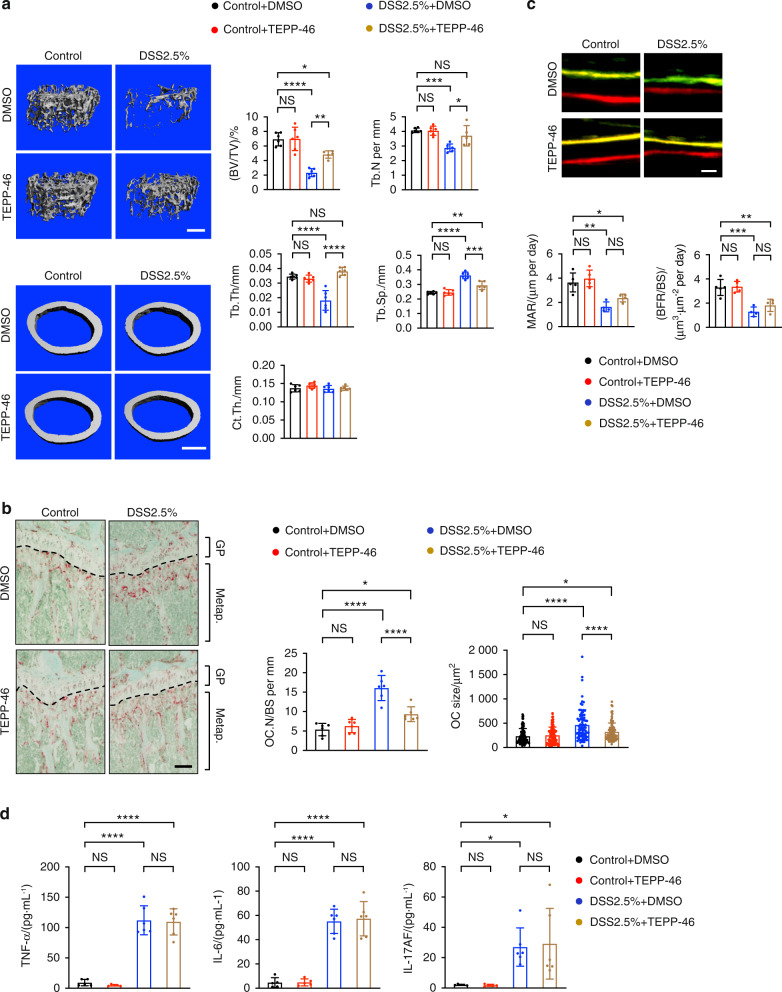
The activation of PKM2 mitigates colitis-induced osteoclastic bone loss. **a** Representative μCT images of the femurs of mice treated with DSS or the control with or without TEPP46. Bone volume per tissue volume (BV/TV), trabecular thickness (Tb.Th), trabecular number (Tb.N), trabecular spacing (Tb.Sp), bone mineral density (BMD), and cortical thickness (Ct.Th) are shown (*n* = 4–6 mice per group). Scale bars represent 0.5 mm. **b** Representative TRAP staining images of tibias in (**a**). TRAP^+^ osteoclast size (OC size) and number (OC.N/BS) are shown. The scale bar represents 200 μm. GP Growth plate, Metap. Metaphysis. **c** Representative dynamic histomorphometry of tibias in Panel (**a**). The mineral apposition rate (MAR) and bone formation rate (BFR) are shown. The scale bar represents 20 μm. **d** Serum levels of TNF-α, IL-6, and IL-17AF from the mice in (**a**) were measured by ELISA. Each dot represents the result of a single mouse. Each dot in OC size represents the result of a single osteoclast. The data are presented as the means ± S.D

We sought to further investigate the effect of TEPP46-mediated activation of PKM2 on osteoclast differentiation in another model of pathological bone loss. Inflammatory bowel disease (IBD) is a chronic inflammatory disease with detrimental skeletal consequences^41^; thus, we used the well-established murine IBD/colitis model of dextran sodium sulfate (DSS)-induced gut inflammation.^42^ Wild-type mice were given DSS in their drinking water for four days, followed by an additional 14 days of regular drinking water. TEPP46 was administered every day for the first five days and then once every two days thereafter. DSS administration resulted in a significant loss of body weight, which peaked on Day 8, and a shortened colon length (Fig. S3). Inflammation was also evident in the colon sections of DSS-treated mice. There were alterations in epithelial structure, severe crypt damage, and mucosal hyperplasia (Fig. S3), confirming DSS-induced colitis. High-resolution μCT imaging revealed a significant reduction in bone mass in the trabecular bone of DSS-treated mice (Fig. 6a). TRAP staining revealed increases in cell size and the number of TRAP^+^ osteoclasts on the bone surface in DSS-treated mice compared to control mice (Fig. 6b). In contrast, concurrent administration of TEPP46 mitigated DSS-induced bone loss (Fig. 6a) and significantly decreased the size and the number of TRAP^+^ osteoclasts on the bone surface (Fig. 6b). Dynamic histomorphometry revealed that TEPP46 administration did not alter bone formation rates, although DSS treatment significantly reduced bone formation rates in control and TEPP46-treated mice (Fig. 6c). DSS-induced colitis and increased serum levels of inflammatory cytokines were observed regardless of TEPP46 administration (Fig. S3 and Fig. 6d), suggesting that TEPP46 treatment did not affect DSS-induced colonic inflammation. These results suggest that the activation of PKM2 by TEPP46 mitigates inflammation-induced bone loss by directly inhibiting osteoclast differentiation and maturation without affecting osteogenesis or inflammation.

## Discussion

During osteoclast differentiation, osteoclast precursors undergo proliferation during the early stages of RANKL treatment.^43–45^ This proliferation is reduced in the later stage, as mononuclear osteoclasts prepare to undergo cell fusion to become large multinucleated mature osteoclasts.^45,46^ Thus, despite undergoing slow or even no proliferation, maturing osteoclasts are expected to maintain the capacity to activate anabolic processes to meet the metabolic demands of increasing in size. It has been observed that aerobic glycolysis, which directs the metabolic fate of glucose toward anabolic processes, is maintained and even increases during osteoclast differentiation.^37^ However, it is not well understood by what mechanism(s) osteoclasts manage their changing metabolic requirements.

**Fig. 7:**
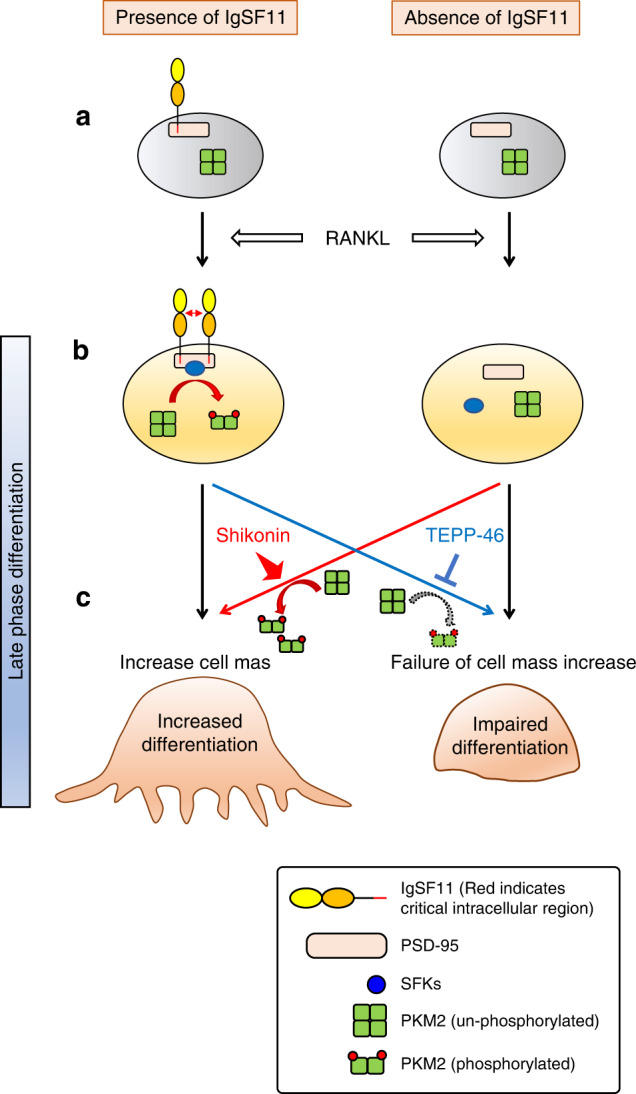
Schematic depicting the role of IgSF11-mediated regulation of PKM2 activity during osteoclast differentiation. **a** PKM2 activity is high in BMMs. RANKL stimulation enhances the expression of IgSF11. **b** IgSF11 mediates the phosphorylation of PKM2 through Src family kinases (c-Src, Fyn, and HcK) in a manner dependent on the C-terminal region. This phosphorylation leads to the inhibition of PKM2 activity. In the absence of IgSF11, the inhibition of PKM2 activity barely occurs, keeping PKM2 activity high. **c** IgSF11-mediated inhibition of PKM2 activity increases osteoclast differentiation. In the absence of IgSF11, osteoclast differentiation is impaired due to a lack of PKM2 inhibition, which can be rescued by the PKM2-specific inhibitor shikonin. Moreover, the PKM2-specific activator TEPP46 inhibits osteoclast differentiation in the presence of IgSF11

In this study, we revealed that IgSF11, a member of the Ig-CAM family, increases osteoclast differentiation by inhibiting PKM2, a key regulator of glycolysis and cell metabolism, by promoting phosphorylation at Y^105^. We also showed that IgSF11 stimulation induced the phosphorylation of PKM2 via the activation of multiple SFKs (c-Src, Fyn, and HcK) through its 75 C-terminal amino acids, which are required for association with PSD-95. Phosphorylation of PKM2 at Y^105^ has been shown to inhibit its enzymatic activity, and a reduction in PK activity is widely believed to promote anabolic processes by shunting glucose metabolites toward the creation of biomass.^17^ Furthermore, PKM2, which is capable of undergoing allosteric regulation, is often expressed in normal proliferating cells and most cancer cells.^47–51^ In this study, we found that proliferating osteoclast progenitors (BMMs) expressed PKM2, and mature osteoclasts maintained PKM2 expression levels. This expression pattern suggests that maturing, nonproliferating osteoclasts possess the capacity to regulate pyruvate kinase activity to shift from energy production to biosynthetic and anabolic pathways. Our data suggest that this regulatory switch via PKM2 operates during osteoclast differentiation and that IgSF11 is a key molecular regulator of PKM2 during osteoclast differentiation (Fig. 7).

We showed that negative regulation of PKM2 during osteoclast differentiation is required for proper osteoclast maturation. However, we found that TAT-Cre-mediated deletion of PKM2 inhibited osteoclast differentiation (Fig. S4). PKM2 deletion has been shown to induce compensatory expression of the constitutively active PK isoform PKM1 in some cell types.^22,52,53^ However, it seems that this compensatory expression does not apply to osteoclasts because the expression of PKM1 remained low even after the deletion of PKM2 (Fig. S4). Given that PKM2 phosphorylation is gradually increased during the late phase of osteoclast differentiation, the active form (i.e., nonphosphorylated form) of PKM2 may be required to mediate the energy generation needed for early-phase osteoclast differentiation. Furthermore, its absence cannot be compensated for by PKM1.

We showed that PKM2 phosphorylation is increased during osteoclast differentiation. As the expression of IgSF11 increases during osteoclast differentiation,^21^ an IgSF11-mediated increase in PKM2 phosphorylation might not be required for the early phase of osteoclast differentiation. However, it may be important for the later phase. we previously revealed that the early stage of differentiation was unaffected in IgSF11^−/−^ cell cultures.^21^ Additionally, we showed that the basal level of phospho-PKM2 was similar in IgSF11^+/+^ and IgSF11^−/−^ BMMs (Fig. 2c), and their proliferation during the early phase of osteoclast differentiation was not affected (data not shown). These results suggest that the increased inhibition of PKM2 activity by phosphorylation at Y^105^ via IgSF11 activation is necessary for full osteoclast differentiation. Using small molecules to specifically modulate PKM2 activity, we showed that controlled PKM2 activity is required for proper osteoclast differentiation and bone metabolism. Although Shikonin has been reported to have an inhibitory effect on p65-NF-κB activation in macrophages,^54^ we found that neither Shikonin nor TEPP46 affected RANKL-induced NF-κB signaling in osteoclasts (Fig. S5), confirming the specificity of these small molecules. Moreover, the importance of PKM2 activity in osteoclasts and bone metabolism was more prominent under pathological conditions. Inflammation causes bone loss due to the induction of multinucleated osteoclasts, and this effect was significantly mitigated by the administration of a PKM2 activator. We found that inflammatory stimuli, such as IL-1β and TNF-α, enhanced the phosphorylation of PKM2 in RANKL-induced osteoclasts (Fig. S6), suggesting that PKM2 activity is negatively regulated by inflammatory stimuli in osteoclasts. TEPP46 treatment did not alter DSS-induced colitis aggravation or osteoblastic bone formation. Additionally, TEPP46 treatment mitigated bone loss induced by RANKL. These results suggest that TEPP46 selectively targets osteoclast differentiation but not inflammation or osteogenesis. It has been reported that TEPP46 inhibits T-cell activation and T-cell-mediated autoimmune responses.^55^ However, a lower dose of TEPP46, which inhibited osteoclast differentiation (Fig. 3b), neither inhibited nor enhanced T-cell activation (Fig. S7). We used a lower dose of TEPP46 in the DSS-induced colitis model (Fig. 6) than a previous report,^55^ and the lower dose of TEPP46 does not seem to affect T-cell activation and inflammation. In the LPS-induced bone loss model, we used the same dose as in a previous report and found comparable production of inflammatory cytokines in control and TEPP46-injected mice (Fig. 5c). Given the differences in the experimental period between EAE (more than two weeks)^55^ and LPS-induced bone loss (five days) (Fig. 5), such a short experimental period may not be sufficient to see TEPP46-mediated anti-inflammatory effects.

Previously, we showed that mutants of IgSF11 in which the intracellular region was truncated, including IgSF11-Mt_353_, failed to rescue the defective osteoclast differentiation in IgSF11^−/−^ cultures, suggesting the role of IgSF11 as a signaling mediator.^21^ Designing a chimeric receptor with the human CD3 extracellular domain and the IgSF11 intracellular region enabled us to induce IgSF11-dependent signals by crosslinking chimeric receptors using anti-human CD3 antibodies. We activated IgSF11-mediated signals in a controlled and synchronized manner with these tools, which made it possible to identify changes in the early signaling profile. Using this approach, we showed that multiple proteins were rapidly phosphorylated at tyrosine residues, suggesting that IgSF11 stimulation leads to the activation of tyrosine kinase(s). Additionally, using an unbiased biochemical approach and mass spectrometry, we identified PKM2 as a major tyrosine-phosphorylated protein downstream of IgSF11 stimulation. The results were obtained using the chimeric receptor and revealed that IgSF11 was a mediator of the signaling necessary for osteoclast differentiation.

IgSF11 was previously shown to interact with PSD-95 via its C-terminal region,^20,21^ and PSD-95 is a known scaffolding protein for multiple kinases, including SFKs.^25^ We hypothesized that IgSF11-PSD-95 signaling complexes induce PKM2 phosphorylation by recruiting kinases and showed that SFKs (c-Src, Fyn, and HcK) mediated IgSF11-induced phosphorylation of PKM2. However, we cannot exclude the possibility of the involvement of other protein tyrosine kinases associated with IgSF11-induced phosphorylation of PKM2. This is because we observed a substantial amount of PKM2 phosphorylation after treatment with the inhibitors, especially dasatinib, which is known to inhibit c-Src, Fyn, and HcK. Further studies will be required to clarify the mechanism by which PKM2 phosphorylation is stimulated by IgSF11.

Interestingly, IgSF11 expression is frequently upregulated in intestinal-type gastric cancers.^56^ Moreover, the expression of IgSF11 has been reported in colorectal cancers, hepatocellular carcinomas, and intestinal-type gastric cancers, and RNAi knockdown of IgSF11 has been reported to hinder the growth of gastric cancer cells.^57^ PKM2 is highly expressed in most cancer cells and promotes tumorigenesis by regulating aerobic glycolysis, which enhances biosynthesis and supports cell proliferation.^58^ It is possible that IgSF11 may have a similar role in cancer growth by modulating the enzymatic activity of PKM2. Thus, IgSF11 may be a possible target for cancer treatment.

In conclusion, we identified PKM2 as a factor in IgSF11-mediated osteoclast differentiation. Previous characterization of the biological function of PKM2 strongly implicates the IgSF11-PKM2 pathway in the regulation of glucose metabolism, and this regulation leads to macromolecule synthesis via the production of numerous intermediate metabolites that are critical for biosynthetic pathways. The identification and elucidation of additional significant regulators of osteoclast differentiation would be helpful in the development of therapeutic strategies for treating skeletal disorders. Taken together, our results identify IgSF11 as a regulator of PKM2-mediated glucose metabolism during the late stages of osteoclast differentiation, and inhibiting this pathway inhibits RANKL-induced bone loss without affecting coupled bone formation in vivo.

## Materials and methods

### Mice

*IgSF11-*knockout (IgSF11^−/−^) mice were generated as described previously.^21^ Homozygous IgSF11^+/+^ and IgSF11^−/−^ littermate mice were generated by intercrossing heterozygous mice. C57BL/6 mice were purchased from the Jackson Laboratory. In each experiment, age- and gender-matched mice were compared. All experimental procedures were performed in accordance with the guidelines approved by the Institutional Animal Care and Use Committee (IACUC) of the University of Pennsylvania.

### Reverse transcription and quantitative real-time polymerase chain reaction (Q-PCR)

Total RNA was extracted using TRIzol reagent (Invitrogen) and reverse transcribed using reverse transcriptase: (Cat.# 18080044; Invitrogen). cDNA was obtained from total RNA and was analyzed by Q-PCR using SYBR green (Applied Biosystems) or QuantStudio3 (Applied Biosystems). For SYBR Green analysis, the sequences of the primers for PKM1 and PKM2 were as follows: PKM1 (forward: GTCTGGAGAAACAGCCAAGG, reverse: TCTTCAAACAGCAGACGGTG) and PKM2 (forward: GTCTGGAGAAACAGCCAAGG, reverse: CGGAGTTCCTCGAATAGCTG).^22^ Mouse 18 S primers (forward: GCCATGCATGTCTAAGTACGC, reverse: TCTGATAAATGCACGCATCC) were used for normalization. For QuantStudio3 analysis, the specific TaqMan probes were mouse IL-2 (Mm00434256_m1) and 18 S (Hs99999901_s1).

### Western blot analysis

Cell cultures were lysed with ice-cold RIPA lysis and extraction buffer (Cat.# 89900; Thermo Fisher) containing a protease and phosphatase inhibitor cocktail (Roche). Mouse tissues (heart and brain) were then washed with PBS and lysed with ice-cold RIPA buffer using a homogenizer. The protein concentrations were determined using a Bradford assay, followed by electrophoretic resolution and transfer to PVDF membranes. Western blotting was then performed with the following antibodies: anti-panphosphotyrosine (4G10 Platinum; Cat.# 05-1050X; EMD Millipore), anti-PKM1 (Cat.# 7067; Sigma–Aldrich), anti-PKM2 (Cat.# 4053; Sigma–Aldrich), anti-Flag: M2 (Cat.# 1804; Sigma–Aldrich), anti-phospho PKM2 Y105 (Cat.# 3827; Cell Signaling Technology), anti-phospho c-Src Y416 (Cat.# 2101; Cell Signaling Technology), anti-c-Src (Cat.# Sc-19; Santa Cruz), anti-phospho JAK2 (Cat.# 3771; Cell Signaling Technology), anti-JAK2 (Cat.# 3230; Cell Signaling Technology), anti-phospho Lyn (Cat.# ab226778; Abcam), anti-Lyn (Cat.# 610003; BD Biosciences) anti-phospho Fyn Y420 (Cat.# MBS9128730; MyBioSource.com), anti-Fyn (Cat.# 4023; Cell Signaling Technology) anti-phospho cAbl (Cat.# 2865; Cell Signaling Technology), anti-phospho HcK (Cat.# ab61055; Abcam), anti-HcK (Cat.# 14643; Cell Signaling Technology), anti-actin (Cat.# sc-47778; Santa Cruz), anti-tubulin (Cat.# sc-5274; Santa Cruz), anti-PSD-95 (Cat.# MMS-5182; Biolegend), and anti-IκBα (Cat.# 9242; Cell Signaling Technology).

### Osteoclast differentiation in vitro

Cells were prepared as described previously.^21^ Briefly, BMMs were induced from whole BMs cultured with M-CSF in a-MEM containing 10% FCS for three days. Osteoclasts were induced from BMMs with M-CSF + RANKL for three days. Osteoclasts were stained using a kit (387A-1KT, Sigma) according to the manufacturer’s instructions. TEPP46 (Cat.# ML-265) and Shikonin (Cat.# 14751-10) were purchased from Cayman Chemical. Anti-human CD3 (HIT3a) antibodies were purchased from Biolegend. L-serine (Cat.# 84959), ribose 5-phosphate (R5P) (Cat.# 83875), and dihydroxyacetone phosphate (Cat.# D7137) were purchased from Sigma. IL-1β (Cat.# 211-11B) and TNF-α (Cat.# 315-01 A) were purchased from Peprotech.

### Measurement of lactate production

BMMs from IgSF11^+/+^ and IgSF11^−/−^ mice were cultured with M-CSF and RANKL for three days. Culture supernatants were harvested every day, and lactate levels in the supernatant were measured using the Lactate Colorimetric/Fluorometric Assay kit (Cat.# K607, BioVision).

### Mass spectrometry

Cell lysates were prepared from anti-hCD3 antibody-stimulated IgSF11^−/−^ hCD3-iFL cultures and passed through phosphoprotein affinity columns (phosphoprotein purification kit, QIAGEN) according to the manufacturer’s instructions. Eluates were fractionated by SDS–PAGE on 4%–12% gradient gels and stained with Coomassie blue. The gel section around the ~55 kD band was cut out. The gel section was subjected to LC–MS/MS analysis by Applied Biomics.

### Retrovirus preparation and transduction

Virus particle preparation and transduction were performed as described previously.^21^ Plat-E packaging cells were transfected with pMX vectors encoding C-terminally FLAG-tagged hCD3-iFL, hCD3-iMt_353_, IgSF11-FL, and IgSF11-Mt_353_ using PEImax (Polysciences). The empty pMX vector was used as a negative control.

### Microcomputed tomography

Femora were harvested, fixed, and scanned as described previously.^21^ For calvaria analysis, bone tissues were harvested by surgical dissection. The tissues were fixed in fixation buffer (Cat.# HT501128, Sigma) overnight, and scans were performed at an isotropic voxel size of 15 µm. The 3D images were then reconstructed to visualize the destructive lesions.

### Bone histology and histomorphometry

For long bones, tibiae were harvested, fixed overnight, and then incubated with 10% EDTA for two weeks for decalcification. Bone sections with a thickness of 10 μm were prepared,^59,60^ followed by TRAP staining. For calvaria, bone tissues were fixed and decalcified, as described previously.^40^ Then, the tissues were sectioned at a thickness of 10 μm using Kawamoto’s film method. The sections were stained with TRAP, and then the area of TRAP^+^ multinucleated cells on the bone surface and the size of the osteoclasts were determined. Quantitative analysis was performed using ImageJ. The measurement of the mineral apposition rate was performed as described previously.^21^

### PKM2 inhibitor and activator injection

For Shikonin administration, seven-week-old male C57BL/6 mice were intraperitoneally injected with 2 mg·kg^–1^ Shikonin or DMSO twice per week for four weeks. For TEPP46 administration, seven-week-old female C57BL/6 mice were intraperitoneally injected with 5 mg·kg^–1^ TEPP46 or DMSO every two days for six weeks. Body weight was measured before every injection.

### Pathological bone destruction by LPS

An animal model of pathologic bone destruction was generated as described previously.^40^ In brief, LPS (12.5 mg·kg^–1^) or PBS was injected into the subcutaneous tissue of nine-week-old male C57BL/6 mouse calvaria. The mice were simultaneously subcutaneously injected with TEPP46 (50 mg·kg^–1^) or DMSO, followed by an additional injection for three days. The mice were then euthanized five days after LPS injection, and serum samples were collected to measure TNF-α (Cat. # 555268, BD Biosciences), IL-6 (Cat. # 555240, BD Biosciences), and IL-17AF (Cat. # 88-8711, Invitrogen) by ELISA. Calvaria were harvested, fixed, and subjected to μCT analysis followed by histomorphometric analysis.

### RANKL-induced bone destruction

A single dose of RANKL (1 mg·kg^–1^) or PBS was injected into the subcutaneous tissue of seven-week-old male C57BL/6 mouse calvaria. The mice were subcutaneously injected with TEPP46 (50 mg·kg^–1^) or DMSO, followed by an additional injection for one day. The mice were euthanized three days after RANKL injection. Calvaria were harvested, fixed, and subjected to μCT analysis followed by histomorphometric analysis.

### DSS-induced colitis

DSS-induced colitis was induced as described previously^42^ with some modifications. Eight-week-old female C57BL/6 mice received 2.5% DSS (molecular mass 36 000~50 000 kD) in their drinking water for four days. This was followed by regular drinking water for 14 days during the recovery period. The mice were intraperitoneally injected with TEPP46 (5 mg·kg^–1^) or DMSO every day for the first five days, followed by injections every two days until the end of the experiment. Body weight was recorded daily. On Day 18 after the initiation of the experiment, the mice were euthanized, and serum samples were collected to measure TNF-α, IL-6, and IL-17AF levels by ELISA. The colon was harvested for histology, and the long bones were harvested for μCT scan and bone histomorphometry analysis. The colon was fixed in 4% paraformaldehyde overnight and then stored in PBS. Tissue processing, wax embedding, sectioning, and H&E staining were performed as described previously.^61^ Colon pathology in all mice was graded blindly using an established histological grading scheme.^61^

### TAT-Cre-mediated PKM2 deletion

PKM2 floxed mutant mice (PKM2^f/f^) were purchased from the Jackson Laboratory (Cat.# 024048). BMMs from PKM2^f/f^ mice were treated with Cre recombinase TAT-cre (Cat.# EG-1001, Excellgen) overnight to generate PKM2-deficient BMMs. The cells were cultured with M-CSF and RANKL for three days.

### In vitro T-cell activation

CD4^+^ T cells were isolated from wild-type mouse spleens using MACS sorting (Cat.# 130-117-043) (Miltenyi Biotec). The isolated CD4^+^ T cells were then plated (1.8 × 10^7^ per well) in 12-well culture plates coated with anti-mouse CD3 (Cat.# 145-2C11) (Cat.# 16-0031-85, eBioscience) and then cultured with anti-mouse CD28 (37.51) (Cat.# 102101, Biolegend) in the presence or absence of TEPP46 (0.5 or 2 μmol·L^–1^) in RPMI medium containing 10% fetal bovine serum for one day. Unstimulated T cells were used as a control.

### Statistical analysis

One-way ANOVA, 2-way ANOVA, or 2-tailed paired Student’s *t* test were used to determine the significance of differences by Prism 9.0 (GraphPad Software). *P* < 0.05 was considered statistically significant.

## Supplementary information

Supplemental information
