# Supplementary material for: IgSF11-mediated phosphorylation of pyruvate kinase M2 regulates osteoclast differentiation and prevents pathological bone loss
Source: Bone Res. 2023 Mar 16;11:17. doi: 10.1038/s41413-023-00251-2 (PMC10020456; doi:10.1038/s41413-023-00251-2)
Supplement: Supplementary file 1 — Supplemental information [file 41413_2023_251_MOESM1_ESM.docx]

**Supplementary Figure 1. Isolation of IgSF11 stimulation-induced phosphoproteins.**

(A) A list of phosphorylated proteins induced by anti-hCD3 antibody stimulation. The major band (~55 kD m.w.) excised from a Coomassie blue-stained gel (Figure 1D) was subjected to mass spectrometry. We set a cutoff score for the exponentially modified protein abundance index (emPAI), which represents the relative quantification of proteins in a mixture, at 2.0 and identified the top three candidate proteins. (B) Sequence of a phospho-PKM2 peptide identified by LC–MS/MS. Mr(expt); experimental m/z transformed to a relative molecular mass, Mr(calc); relative molecular mass calculated from the matched peptide sequences. (C) Vimentin is not phosphorylated by IgSF11. Total cell lysates were prepared from anti-hCD3 antibody-stimulated IgSF11^-/-^ BMMs retrovirally transduced with hCD3-iFL, followed by culture with M-CSF + RANKL for two days, and immunoprecipitated with anti-vimentin antibody. Western blotting was performed with the indicated antibodies.

**Supplementary Figure 2. Shikonin- or TEPP46-treatment has no major side effects.**

(A and B) Body weight changes of mice treated with Shikonin or TEPP46 were shown. The percentage change in the body weight of mice treated with Shikonin for four weeks (A) or TEPP46 for six weeks (B). Control mice were injected with DMSO. (C and D) Histological analysis of Heart, Liver, Kidney and Spleen of mice treated with Shikonin for four weeks (C) or TEPP46 for six weeks (D). Magnified view of the boxes were shown in the bottom. Scale bars represent 100 μm. Data is presented as the means ± S.D.

**Supplementary Figure 3. DSS-induced colitis.**

(A) Percentage change in the body weight of mice treated with or without 2.5% DSS for four days, followed by a recovery period. The mice were injected with TEPP46 or DMSO every day for the first five days, followed by injection every two days until the end of the experiment. Data is presented as the means ± S.D. (B) Representative images of colons from mice in panel (A). Colon lengths are shown. (C) Representative H&E-stained sections of colon from mice in panel (A). Histological scores are shown. Each dot represents the result of a single mouse.

**Supplementary Figure 4. PKM2 deletion inhibits osteoclast differentiation.**

PKM2^f/f^ mouse-derived BMMs treated with or without TAT-Cre were cultured with M-CSF + RANKL for three days. (A) Protein expression levels of PKM isoform 1 and isoform 2 in PKM2-deleted osteoclasts were determined by western blotting. (B) Representative micrographs of TRAP staining. The frequency of TRAP^+^ multinucleated cells (3 nuclei or more per cell) is shown. Scale bars represent 100 μm. Each dot represents technical replicates. The results are representative of three independent experiments.

**Supplementary Figure 5. Shikonin or TEPP46 does not affect RANKL-induced NF-kB activation.**

Wild-type mouse-derived BMMs were treated with M-CSF and RANKL for two days to induce pre-osteocasts, then treated with Shikonin (+ and ++ indicate 5 and 120 nM, respectively) or TEPP46 (+ and ++ indicate 0.07 and 1.9 μM, respectively) in the presence of RANKL for 10 min. Whole cell lysates were used for western blotting with the indicated antibodies.

**Supplementary Figure 6. PKM2 activity is negatively regulated by inflammatory stimuli.**

Wild-type mouse-derived BMMs were treated with M-CSF and RANKL for two days to induce pre-osteoclasts, then treated with IL-1β (20 ng/ml) or TNF-α (20 ng/ml) in the presence of RANKL for one day (D3). BMMs were used as a negative control (D0). Whole cell lysates were used for western blotting with the indicated antibodies.

**Supplementary Figure 7. The effect of TEPP46 on T cell activation.**

CD4^+^ T cells isolated from wild-type mouse spleen were cultured on the plates coated with anti-CD3 antibody with anti-CD28 antibody in the presence or absence of TEPP46 (0.5 and 2 μM) for one day. Total RNA was then isolated from T cells, and the expression levels of IL-2 were analyzed by Q-PCR. Data is presented as the means ± S.D. Each dot represents technical replicates.
